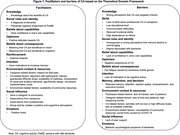# Barriers and Facilitators to Participation in Cognitive Activities in Persons with Mild Dementia: A Qualitative Study Using the Theoretical Domain Framework

**DOI:** 10.1002/alz70860_098119

**Published:** 2025-12-23

**Authors:** XUE Dandan, Jiaying Li, Meiqing Sheng, Wenwen Qi

**Affiliations:** ^1^ Department of Nursing, Gongli Hospital of Shanghai Pudong New Area, Shanghai, China; ^2^ School of Nursing, Johns Hopkins University, Baltimore, MD, USA; ^3^ Shanghai Mental Health Center, Shanghai Jiao Tong University School of Medicine, Shanghai, China

## Abstract

**Background:**

Cognitive interventions can delay cognitive decline in persons with mild dementia (PwMD), but various obstacles often impede their participation. Limited research has examined factors that facilitate or hinder their engagement in cognitive activities. This study utilizes the Theoretical Domain Framework (TDF) to identify the barriers and facilitators influencing cognitive activities participation among PwMD.

**Method:**

This qualitative study was conducted at a geriatric clinic in a hospital in Shanghai and involved individual semi‐structured interviews with 12 PwMD and their family caregivers. An interview guide with open‐ended questions was used to facilitate the interviews conducted via Tencent Meeting. The interviews were audio‐recorded, transcribed verbatim, and analyzed through directed content analysis.

**Results:**

Twelve out of the fourteen TDF domains related to facilitators and barriers were identified (Figure 1), including knowledge, skills, social roles and identity, beliefs about capabilities, optimism, beliefs about consequences, reinforcement, intention, memory, attention, decision, environment context and resources, social influence, and emotions. The low confidence of PwMD in their abilities, and the presence of psychiatric and behavioral symptoms, acted as barriers. Conversely, caregivers’ positive attitudes and strong beliefs in the benefits of cognitive activities served as motivators. Challenges related to memory, attention, and decision, along with reduced functional ability, increased PwMD's dependence on others for support in cognitive activities. The assistance and supervision from caregivers served as enablers, while the inadequate caregiver support, heavy caregiving burdens, and time constraints faced by caregivers posed obstacles. Additionally, environmental context and available resources significantly influenced level of participation. Engagement was enhanced by activities that were appropriately challenging, age‐appropriate, visually appealing, aligned with individual interests, practical to implement, and supported by training materials.

**Conclusion:**

This study highlights the complex factors facilitate or hinder cognitive activities participation in PwMD and provides valuable insights for designing effective, engaging cognitive interventions to help prevent cognition decline in this population.